# Differential dietary intake and contribution of ultra-processed foods during pregnancy according to nutritional status

**DOI:** 10.3389/fnut.2024.1400513

**Published:** 2024-06-14

**Authors:** Adriana Granich-Armenta, Alejandra Contreras-Manzano, Alejandra Cantoral, Dirk L. Christensen, Joaquín A. Marrón-Ponce, Laura Ávila-Jímenez, Ivonne Ramírez-Silva, Juan A. Rivera Dommarco, Louise G. Grunnet, Ib C. Bygbjerg, Héctor Lamadrid-Figueroa

**Affiliations:** ^1^Center for Population Health Research, National Institute of Public Health, Cuernavaca, Mexico; ^2^CONAHCYT, Nutrition and Health Research Center, National Institute of Public Health, Cuernavaca, Mexico; ^3^Health Department, Universidad Iberoamericana, Mexico City, Mexico; ^4^Department of Public Health, Section of Global Health, University of Copenhagen, Copenhagen, Denmark; ^5^Nutrition and Health Research Center, National Institute of Public Health, Cuernavaca, Mexico; ^6^Instituto Mexicano del Seguro Social, Cuernavaca, Mexico; ^7^Clinical Prevention Research, Steno Diabetes Center Copenhagen, Herlev, Denmark

**Keywords:** ultra-processed foods, hemoglobin levels, NOVA classification, nutritional status, pre-gestational BMI

## Abstract

**Introduction:**

Frequent consumption of ultra-processed foods (UPFs) during pregnancy is linked to excess intake of added sugar, fat, and sodium and inadequacy of several micronutrients. Diet quality during pregnancy should be maximized as inadequate levels of key nutrients and excessive intake of energy and added sugar might influence mother–child health. We aimed to estimate the contribution (% of total calories) of ultra-processed products to the total energy intake by pre-gestational body mass index (BMI) categories and Hb status during pregnancy in participants from the MAS-Lactancia Cohort.

**Methods:**

Pre-gestational weight, hemoglobin levels, 24-h dietary intake recall interviews, and sociodemographic data were collected during the second and third trimesters of pregnancy. Reported consumed foods were categorized using the NOVA classification, and the contribution of calories from each NOVA category was estimated using the Mexican Food Database. We estimated medians and interquartile ranges (p25 and p75) for dietary intake and energy contributions. The comparison of intake between the second and third trimesters was done using the Wilcoxon test. In addition, a quantile regression model with an interaction between pre-gestational BMI and Hb levels status in tertiles over the percentage of energy from UPFs was adjusted by age and socioeconomic status.

**Results:**

The contribution to total energy intake from UPFs was 27.4% in the second trimester and 27% in the third trimester (with no statistical difference). The percentage of energy intake from UPFs was higher in women who started pregnancy with obesity and presented the lowest levels of Hb (1st tertile), 23.1, 35.8, and 44.7% for the 25th, 50th, and 75th percentiles, respectively, compared to those with normal BMI and the highest tertile of Hb levels: 18, 29.0, and 38.6% for the 25th, 50th, and 75th percentiles, respectively.

**Conclusion:**

In conclusion, UPF intake in pregnant women is similar to the general population and was higher for those with pre-gestational obesity and the lowest tertile of Hb levels. UPF contributes also to sugar, saturated fat, and sodium, which may adversely affect the health of mothers and their offspring.

## 1 Introduction

Nutritional status in women of reproductive age is crucial for mother and child health, having a major influence on the short- and long-term health of both (1–3). The combined prevalence of overweight and obesity in Mexican women was 75% in 2021, and it is estimated that 41% of Mexican women of reproductive age live with obesity (4). Another recent public health concern in Mexico is anemia during pregnancy, with 35% of pregnant women having serum hemoglobin concentrations below 11 g/dL (5, 6).

Ultra-processed foods (UPFs) are typically characterized as being high in energy density, added sugars, trans and saturated fats, and sodium while being low in fiber and key micronutrients (7, 8). Diets with nutrient-poor, high-energy-density foods, such as UPFs, are taking over fresh and minimally processed foods that are the basis of traditional healthy diets (9). It has been reported that Mexico is the leading country in UPF consumption in Latin America (214 kg/*per capita* annually retail sales in 2013) (10), with close to 30% of the total energy intake contributed by UPF in Mexican adults (11). Thus, on average, the quality of the Mexican diet is low and may be contributing to excessive weight gain and anemia during pregnancy (12).

There is consistent evidence of a higher risk of adverse health outcomes in children and adults associated with the consumption of UPF worldwide (13). Recent literature reported studies of the association between UPF consumption in pregnant women and gestational diabetes mellitus (14), excessive gestational weight gain (15, 16), hypertensive disorders of pregnancy (17), and shortened gestation (18).

Therefore, the aim of this study was to characterize the intake of UPFs in pregnancy as a percentage of total calories consumed and estimate the distribution of calories consumed by UPFs according to the pre-gestational BMI and the levels of Hb during pregnancy. Finally, we estimated that UPF consumption was different according to the pre-gestational BMI status and the levels of Hb during pregnancy.

## 2 Materials and methods

### 2.1 Study design and population

For this study, we used data from the MAS-Lactancia birth cohort. Participants in the birth cohort were pregnant women affiliated with the Mexican Social Security Institute (IMSS, acronym in Spanish) located in Morelos, Mexico, which provides health services and social security to private/formal employees and their families. The women were 18–39 years old and recruited during the 16th to 22nd gestational weeks of singleton pregnancies and followed up from week 34 to the pregnancy resolution. More detailed information about the MAS-Lactancia cohort is available elsewhere (19). This project was approved by The National Institute of Public Health (IRB project number 1281 and approval number 1646). All participants who signed the informed consent before data collection during the period from March 2016 to December 2020 were included.

### 2.2 Sample

We analyzed two sub-samples according to the availability of the main variables (diet, nutritional status, height, weight, and hemoglobin levels). We included 660 participants to analyze diet, maternal weight, and anemia. The analytical sample consists of a subsample of 660 participants with diet available and another subsample of 346 with diet and hemoglobin levels available. We used information from the second and third trimesters and pre-gestational BMI data.

### 2.3 Dietary information

The diet information was collected using a 24-h recall Multiple-Pass Method (20). The interview was done randomly on different weekdays and weekends in the sample. We estimated the macro and micronutrient intakes using the same methodology as the 24-h recall analysis in the National Health and Nutrition Survey, 2016 (ENSANUT) (21). To obtain the 24-h recall diet information, we used the nutrient retention factor (NRF) to improve the accuracy of the dietary intake estimation. It was estimated in 4 phases: Phase 1.- Review and correction of all the 24-h recall steps (20); Phase 2: Estimation of food intake in grams; Phase 3: Processing. Estimation of energy and nutrients according to the Mexican Food Database version 18.1.1 (BAM (acronym in Spanish: Base de datos de los Alimentos Mexicanos) in which 1978 foods, recipes, and drinks have been listed (22). The NRF was estimated following the United States Department of Agriculture (USDA), Bergstrom and Bognar reference (23–25). In case the reported food information was not available, we used the NRF average value, the NRF group-specific, and the compilation of the European Food Information Resource (EuroFIR) (26); Phase 4: Energy and nutrient data cleaning. We included nutrients, energy, carbohydrates, total fat, saturated fat, sugars, sodium, potassium, calcium, folic acid, vitamin B12, vitamin B6, vitamin C, and iron content. We aggregated the energy and food/ingredient of the nutrient content at the individual level. The review of dietary intake reported plausible values; the equations were developed by the Institute of Medicine (IOM) according to body mass index (BMI (kg/m^2^), normal weight, overweight or obese) and age group (21).

Outliers were identified based on the relationship between each nutrient intake and the average requirements. For both macronutrients and micronutrients, according to the proportional distribution of each nutrient, standard deviation values <−3 and > +3 were considered outliers or extreme values according to pregnancy and age groups. Pregnant women who reported an intake of fewer than 500 kilocalories and more than 5,000 kilocalories were excluded from the analysis (21). We also include the intake of supplements (specifically iron and folic acid) in the estimations, as they are regularly prescribed.

The percentage of energy from ultra-processed foods to total dietary energy was estimated after obtaining the detailed diet information and having the food list and ingredients disaggregated from the 24-h recall; we classified foods and beverages according to the industrial processing to preserve, extract, or modify their characteristics using the NOVA approach (8). The diet exposure variables were determined following the NOVA approach, which is classified into four groups: (1) non-processed or minimally processed foods, (2) culinary ingredients, (3) processed foods, and (4) UPFs. After having classified the food and beverages, we estimated the contribution from each food and beverage by NOVA group (1, 2, 3, and 4) to the total energy intake, and then we ranked each food and beverage by NOVA group to obtain the food and beverage that most contributed to the energy intake.

### 2.4 Nutritional status

For this analysis, we considered two variables to classify nutritional status, pre-gestational BMI status, and Hb levels in the second and third trimesters. Pre-gestational weight was obtained from the IMSS medical files self-reported at their first medical consultation. Pre-gestational BMI was calculated and categorized into three categories: normal pre-gestational BMI (≥18.5–24.9 kg/m^2^), overweight (≥25–29.9 kg/m^2^), and obesity (≥30 kg/m^2^). We obtained information on Hb levels from each participant’s clinical files, which were analyzed at the IMSS laboratory hospital (UniCel DxC 600/800 SYNCHRON) and recorded in the electronic medical system. The township of residence determined the altitude adjustment for the hemoglobin level, and all hemoglobin levels were adjusted by altitude. Then, we classified anemia during pregnancy according to the WHO guidelines on hemoglobin levels <11 gm/dl (27).

### 2.5 Co-variables

The information about maternal age, years of schooling [education level: elementary school (6 years), high school (12 years), and undergraduate (more than 16 years)], socioeconomic status (Household Wealth Index), marital status (single/married), and employment were obtained from medical records and questionnaires within the MAS-Lactancia cohort data.

### 2.6 Statistical analysis

Continuous variables (age, schooling, and Hb) were estimated as means ± standard deviations, while categorical variables (socioeconomic status, occupation, marital, and nutritional status) were estimated as frequencies and proportions. For dietary variables, we estimated medians and interquartile ranges (p25 and p75) as their distribution was skewed. We compared the intake between the second and third trimesters using the Wilcoxon test.

As dietary data were skewed, the associations were evaluated using a quantile regression. For these models, the outcome is the relationship between a set of predictor variables and the target variable quantile (50th percentile is the most common one) (28). When the median is used instead of the mean to describe the link between the variables, it has advantages over conventional least squares regression, including that the models are more robust or less sensitive to outliers (28, 29). Additionally, it requires no assumptions about the model’s distribution or homoscedasticity (29). In terms of the exposure variables, the Hb levels were divided into three categories based on tertiles. We opted for tertiles instead of simply classifying women as anemic or non-anemic because it is widely recognized that pregnant women with moderate-to-severe anemia face a greater risk of adverse outcomes compared to those with mild anemia (30). Moreover, elevated maternal Hb levels have been linked to adverse maternal and infant outcomes such as gestational diabetes mellitus, preterm birth, low birth weight, and fetal death (31–33).

To avoid the assumption of linear relationship or fixed functional ways, the independent variables were categorized as tertials with the outcome variables, therefore providing the model’s flexibility alongside ease of interpretation. Covariates included in the quantile model were socioeconomic level. Finally, we generated a quantile regression model with an interaction between pre-gestational BMI (normal, overweight, and obesity) and tertiles of hemoglobin levels status over the percentage of energy from UPF (25th, 50th, and 75th percentiles). Normal pre-gestational BMI and Hb levels in the third tertial (the highest) were taken as the reference. We considered a significance level of *p* < 0.05 for the tests and regressions and *p* < 0.1 for the interaction term. All data were analyzed using the Statistical Package Stata software version 14.0.

## 3 Results

The sociodemographic characteristics at enrollment are presented in Table 1. We analyzed 660 participants included in the study in the second and third trimesters of pregnancy with a median gestational age of 20 (IQR 18–24) and 34 weeks (IQR 34–35), respectively. The mean age was 26.4 years old, and the mean schooling was 12.9 years. 50.7% of the participants work in the formal sector. The pre-gestational nutritional status was 58, 31, and 11% for normal (BMI <25 Kg/m^2^), overweight (BMI ≥25 Kg/m^2^ & BMI < 30 Kg/m^2^), and obesity (BMI >30 Kg/m^2^) categories, respectively. It is noteworthy that in the third trimester, these proportions change dramatically to 17.1, 48.7, and 34.2% for normal, overweight, and obesity categories, respectively. On average, participants had a gestational weight gain of 10.3 Kg. Regarding hemoglobin levels, the mean adjusted for altitude levels were 12.3 g/dL and 11.9 g/dL for the second and third trimesters, respectively.

**Table 1:** General characteristics of pregnant women participants (*n* = 660).

	Mean (SD) or frequency (%)
<Age (years)^a^	26.4 (5.02)
Sociodemographic	
Schooling (years)^a^	12.9(3.00)
Socioeconomic Status	
Low	212 (32.1)
Medium	221 (33.5)
High	227 (34.4)
Occupation	
Housewife	234 (35.6)
Informal-student	90 (13.7)
Formal	334 (50.7)
Marital Status	
Single	92 (13.9)
Married - unmarried union	566 (86.1)
Nutritional status	
Pregestational BMI categories	
Normal	347 (58)
Overweight	187 (31)
Obesity	68 (11)
BMI 2nd trimester	
Normal	278 (43.1)
Overweight	261 (40.5)
Obesity	106 (16.4)
BMI 3rd trimester	
Normal	39 (17.1)
Overweight	111 (48.7)
Obesity	78 (34.2)
Gestational weight gain (Kg)	10.3 (4.06)
Hemoglobin Levels (g/dL) 2nd trimester^b^	12.3 (1.2)
Hemoglobin Levels (g/dL) 3rd trimester^b^	11.9 (0.9)

a Means and standard deviations for continuous variables and frequencies.

b Hemoglobin levels are only available in 396 participants.

### 3.1 Diet contributions

We evaluated the daily intake of macronutrients and micronutrients during the second and third trimesters of pregnancy. Medians (interquartile range) of macro and micronutrient daily intake and contribution to the diet are shown in Table 2. The median energy intake in 24 h at enrollment (2^nd^ trimester) was 2,135.3 kcal, with the distribution from carbohydrates, proteins, and total fats of 56.4, 13.1, and 31.3%, respectively.

**Table 2:** Description of diet during pregnancy.

Macro/micronutrients	Daily intake 2nd trimester	Daily intake 3rd trimester	*p*-value^*^
	p50 (p25, p75)	p50 (p25, p75)	
Energy (kcal)	2135.3 (1685.5, 2759.9)	2385.2 (1836.1, 2992.1)	< 0.001
Macro Nutrients			
Carbohydrates (g)	293.5 (231.5, 383.6)	335.1 (253.2, 440.7)	< 0.001
Carbs % of energy	56.4 (49.8, 63)	56.3 (49.9, 64.5)	0.449
Fiber(g)	23.1 (16.8, 33.4)	26.7 (18.9, 35.4)	0.002
Total Sugar(g)	125.1 (90, 164.9)	145 (101.8, 193.1)	< 0.001
Total sugar (%)	23.43 (21.35, 23.89)	24.31 (22.17, 25.8)	0.38
Proteins(g)	71.3 (53.7, 96.5)	78.5 (58.9, 107.3)	0.005
Protein % of energy	13.1 (11.1, 15.8)	13.3 (10.9, 15.8)	0.783
Total Fats (g)	74 (51.8, 101.9)	81 (58.7, 117.7)	0.009
Fat % of energy	31.3 (26.2, 37.2)	31.2 (26.2, 37.3)	< 0.001
Saturated Fats (g)	29.1 (19.7, 41.1)	30.9 (21.1, 44.4)	0.055
Saturated Fats (%)	12.26 (10.51, 13.4)	11.65 (10.34, 13.35)	0.01
Micronutrients			
Total Iron(mg)	57.5 (16.4, 82.2)	49.8 (19.7, 83.7)	0.508
Iron Hem(mg)	0.61 (0.26, 1.2)	0.75 (0.4, 1.4)	0.018
Iron no-Hem(mg)	12.7 (8.6, 19.7)	15.4 (10.4, 21.7)	< 0.001
Folate DFE (mcg)	555.7 (376.7, 828.5)	661 (462.8, 1065.8)	< 0.001
B6 Vitamin (mg)	2.2 (1.4, 4.4)	3.2 (1.7, 5.5)	< 0.001
B12 Vitamin (mcg)	4.7 (2.4, 8.1)	6 (3.1, 10.2)	0.003
Vitamin C (mg)	191.8 (96.6, 350.2)	207 (129.6, 336.7)	0.053
Calcium (mg)	1230.5 (927.1, 658.3)	1287.9 (937.2, 1897.2)	0.086
Potassium (mg)	2,950 (2178.9, 3907.5)	3310.9 (2342.1, 4547.5)	< 0.001
Sodium (mg)	2885.4 (2007.8, 3885.1)	3158.3 (2083.9, 4,638)	< 0.001

^*^Statistical differences were determined using the Wilcoxon test.

The medians of fiber (23.1 g), total sugars (125.1 g), sodium (2885.4 mg), saturated fat (29.1 g), and micronutrients total intake consumed during the 2^nd^ trimester were total iron (57.5 mg), hem-iron (0.61 mg), non-hem-iron (12.7 mg), folate DFE (555.7 𝜇g), B6 vitamin (2.2 mg), B12 vitamin (4.7 mg), vitamin C (191.8 mg), potassium (2,950 mg), and calcium (1230.5 mg). On the other hand, the median estimation of 24 h energy intake at follow-up (3^rd^ trimester) was 2385.2 kcal with almost the same distribution of macronutrients as the percentage of energy intake. The intake of potassium, B12, B6, folate, and non-hem-iron was statistically higher in the third trimester. However, we did not identify significant differences within the daily intake of total iron, iron hem, B12 vitamin, Vitamin C, and calcium between the second and third trimesters. It is important to highlight that even saturated fat decreased from the second to the third trimester; in both trimesters, only 25% of the sample consumed less than 10%, which is the maximum recommended. In the case of sodium, the intake increases from the second to the third trimester, and also only 25% reported consuming 2000 mg/d or less (the amount recommended by WHO).

The median total contribution for energy, total sugar, saturated fat, and sodium intake during the second and third trimesters of the foods classified according to NOVA are shown in Table 3. UPFs (NOVA 4), on average, contribute to 27, 29, 26.5, and 31% of total calories, total sugar, saturated fat, and sodium, respectively (Table 3). The most consumed foods from the NOVA 1 group were traditional tortillas representing 52.5%, followed by milk 12.7%, chicken 9%, beef 7.2%, banana 2.7%, and oatmeal 1.8%. NOVA 2 were vegetable oils (43.3%), sugar (29.7%), brown sugar cane (piloncillo) (4.2%), animal lard (3.9%), butter (2.3%), and vegetable lard (1.4%). NOVA 3 were cheeses (51.7%), corn flour (14.7%), cow’s milk acid cream (8.2%), condensed evaporated milk (6.7%), tortilla, toast [corn flour (MASECA)] (5.5%), and dry-salted meat (cecina) (4.7%). The NOVA 4 (UPFs) most consumed foods were sweet bread, white bread, cookies, cakes and donuts (49.7%), breakfast cereals (6.1%), industrialized juices and soda (6.1%), pizza (5.1%), corn chips (fritters) (5.1%), and candies covered with chocolate (3.5%).

**Table 3:** Consumed food that contributed the most to energy intake by the NOVA group.

Group	Trimester	% Contribution to total energy intake p50 (p25, p75)	% Contribution to total sugar intake p50 (p25, p75)	% Contribution to total saturated fat intake p50 (p25, p75)	% Contribution to Sodium intake p50 (p25, p75)	Tops foods represented by the NOVA group						
NOVA 1	2nd trim	51 (40.9, 62.9)	41.9 (28.3, 59.4)	34.8 (20.4, 54.9)	15.7 (9.6, 25.7)	Food	Tortilla (Traditional, Nixtamalized Corn)	Milk	Chicken	Beef	Banana	Oatmeal
	3rd trim	53.7 (42.6, 63.3)	42.40 (26.92, 58.72)	37.77 (23.45, 52.41)	15.61 (9.75, 25.35)	Contribution	52.5	12.7	9	7.2	2.7	1.8
NOVA 2	2nd trim	10.4 (4.8, 16.8)	0.1 (0, 0.9)	4.1 (1.1, 9)	27.5 (13.3, 45)	Food	Vegetable oils	Sugar	Brown Cane Sugar (Piloncillo)	Animal’s Lard	Butter	Vegetable Lard
	3rd trim	11 (5.4, 17.3)	0.1 (0, 1.1)	4.2 (1.6, 9.1)	28.6 (15.7, 41.9)	Contribution	43.3	29.7	4.2	3.9	2.3	1.4
NOVA 3	2nd trim	3.6 (0.12, 11.1)	18.5 (3.9, 31.4)	19.4 (4.2, 39.6)	13.1 (3.6, 29)	Food	Cheeses	Corn Flour	Cow’s milk acid cream	Condensed evaporated milk	Tortilla/Toasts [Corn Flour (MASECA)]	Dry-Salted meat (cecina)
	3rd trim	3. 3(0.3, 9.5)	16.4 (4.3, 29.5)	20 (4.9, 38)	14 (4.1, 27.5)	Contribution	51.7	14.7	8.2	6.7	5.5	4.7
NOVA 4	2nd trim	27.4 (17.5, 38)	29 (13.4, 46.5)	26.3 (9.5, 44.2)	30.6 (13.8, 44.6)	Food	Sweet Bread, White Bread, Cookies, Cakes, and Donuts	Breakfast Cereals	Industrialized Juices and Soda	Pizza	Corn Chips (fritters)	Candies Covered with Chocolate
	3rd trim	27 (17.3, 37)	28.8 (14, 48.9)	27.1 (11.6, 43.1)	31.7 (18.2, 46.1)	Contribution	49.7	6.1	6.1	5.1	5.1	3.5

Table 4 presents the results of the quantile regressions for the prediction of the 25th, 50th, and 75th percentiles of contribution to energy from the intake of UPF or NOVA 4. The Hb levels were stratified into three categories defined by tertials: T1 mean 11.0 g/dL (range 6.2–11.7), T2 mean 12.2 g/dL (range 11.8–12.7), and T3 mean 13.4 g/dL (12.8–18.9). There is a tendency for the distribution of energy intake from UPF to be higher as pre-gestational BMI categories increase and Hb tertiles decrease. Highlighting in those women that started the pregnancy with obesity (BMI > 30 kg/m^2^) and in the lowest tertile of Hb (≤ 11 g/L), there was a statistically higher intake of UPF for percentiles 50th and 75th. These women had an intake of 35.8% (IQR 27.4–44.3%) and 44.7% (IQR 31.5–57.9%) of energy consumed by UPF, respectively.

**Table 4:** Quantile regression models for the percentiles 25th, 50th, and 75th of the percentage of energy intake from UPFs according to the pregestational BMI and Hb levels (*N* = 346).

	Percentile 25th				Percentile 50th				Percentile 75th			
	Predicted percentage	Standard error	95% intervals		Predicted percentage	Standard error	95% intervals		Predicted percentage	Standard error	95% intervals	
Pregestational BMI												
Normal BMI (*n* = 198)	16.6	1.5	13.8	19.5	27.9	1.6	24.8	31.1	37.8	1.8	34.2	41.4
Overweight (*n* = 104)	19.9	2.1	15.7	24.1	28.6	1.9	24.9	32.2	40.2	2.8	34.6	45.8
Obesity (*n* = 44)	20.1	3.4	13.5	26.7	29.0	2.7	23.7	34.3	37.6	4.5	28.8	46.4
Hb levels												
1st tertile (*n* = 115)	17.7	2.6	12.7	22.7	29.4	1.5	26.4	32.4	39.1	3.0	33.3	44.9
2nd tertile (*n* = 101)	17.0	1.8	13.5	20.4	27.5	2.6	22.4	32.5	38.2	2.6	33.2	43.3
3rd tertile (*n* = 130)	19.2	1.6	16.0	22.3	27.8	1.6	24.6	31.1	38.1	2.1	34.0	42.3
Pregestational BMI^*^tertiles Hb levels												
Normal BMI *1st tertile Hb (*n* = 69)	15.6	3.0	9.8	21.4	27.8	2.0	23.8	31.8	36.8	3.7	29.7	44.0
Normal BMI *2nd tertile Hb (*n* = 56)	15.6	2.1	11.5	19.6	26.7	3.9	19.1	34.4	37.8	3.2	31.5	44.0
Normal BMI *3rd tertile Hb (*n* = 73)	18.4	2.4	13.6	23.1	29.0	2.2	24.7	33.3	38.6	2.7	33.3	44.0
Overweight*1st tertile Hb (*n* = 31)	19.5	4.8	10.1	28.8	29.8	2.5	24.9	34.8	41.1	5.9	29.5	52.6
Overweight*2nd tertile Hb (*n* = 33)	19.1	4.1	11.0	27.2	29.2	3.9	21.6	36.7	39.1	4.5	30.3	47.9
Overweight*3rd tertile Hb (*n* = 40)	20.8	2.2	16.5	25.2	27.0	3.3	20.6	33.3	40.3	3.9	32.7	47.9
Obesity*1st tertile Hb (*n* = 15)	23.1	8.1	7.3	38.9	**35.8**	**4.3**	**27.4**	**44.3**	**44.7**	**6.7**	**31.5**	**57.9**
Obesity*2nd tertile Hb (*n* = 12)	18.2	3.5	11.3	25.2	26.9	6.7	13.8	39.9	38.4	11.7	15.5	61.4
Obesity*3rd tertile Hb (*n* = 17)	18.9	4.4	10.2	27.5	24.6	3.0	18.8	30.4	30.6	4.7	21.4	39.9

^*^Models adjusted by maternal age, SES, and total energy intake. Blood values represents statistically significant differences (*p*<0.05).

## 4 Discussion

This study particularly studied the intake of UPF, including the distribution (median and interquartile range) and the contribution to total calories. We showed that pregnant women have a similar intake of UPF (27% of total calories) as the rest of the Mexican population (30% of total calories) (11), and also agrees with a previous report in a Mexican birth cohort (27.9% of total calories) (34), which can reflect that food quality did not improve according to the gestational status.

Diet quality declines when UPF consumption is increased during pregnancy (35). Our findings indicate that UPFs (NOVA 4) contribute 27, 29, 26.5, and 31% of total calories, total sugar, saturated fat, and sodium, respectively. Previous studies involving American, Norwegian, Brazilian, and Mexican pregnant women have estimated the proportion of UPF in total energy intake, revealing a wide range: over 50% in American women (16, 36), nearly twice the amount found in our study; 46% in Norwegian women; 27.9% in Mexican women; and 20.9% in Brazilian women (34, 37, 38). Furthermore, our analysis highlights the contribution of sodium, saturated fat, and sugar from UPF to the overall nutrient intake during pregnancy.

Furthermore, UPF usually contains some sugar substitutes and other additives that alter intestinal function, interfere/or decrease the bioavailability and absorption, especially of iron, as well as compounds such as acrylamide, which is usually used for the UPF industrialization have been associated with a decrease in hemoglobin biomarkers (39).

Specifically, the highest energy contribution consumed by UPFs (35.8-p50th to 44.7%-p75th) were those with the combination of pre-pregnancy obesity (BMI > 30 kg/m2) and the lowest Hb levels during pregnancy. A previous study in a US cohort showed that the lowest dietary quality among pregnancy was seen in women with pre-pregnancy obesity (40).

Although we did not consider weight gain in the estimations, we observed that average weight gain was 10 kg, with a difference in gestational weight gain only between normal pre-gestational BMI and pre-gestational overweight women (1.2 Kg more in the first group). This could reflect a decline in the normal BMI category from 58% pre-pregnancy to 17% at the end of pregnancy. Concurrently, obesity doubles, rising from 16.4 to 34.2%. In comparison, a study from Brazil estimated that each extra 1% of total calories from UPF led to an increase of 4.17 g/week in the third trimester (15). In addition, a study conducted on US women reported that gestational weight increased by 1.33 kilograms per percentage point, a higher contribution of the UPF to total energy. The same study reported that UPF consumption increases neonatal body fat (16).

Furthermore, a recent meta-analysis identified that maternal consumption of UPF-rich diets was associated with an increased risk of gestational diabetes mellitus and preeclampsia (41). More importantly, pre-gestational UPF consumption could increase the risk of gestational diabetes mellitus (42). Additionally, specific products belonging to UPF categories, such as industrial sweets, bakery products, and pastries, increase the odds of having a small-for-gestational-age newborn (43). Soft drink consumption (>7 times/week) also appears to be a risk factor for gestational hypertension (44). All this evidence underscores the importance of limiting UPF consumption before and during pregnancy to improve maternal and neonatal health.

The strength of this study is that it is nested as a secondary analysis from a cohort, which allows us to have repeated (in both trimesters) measures of Hb and diet. We acknowledge several limitations to this study. First, the included participants were from a secondary-level hospital, i.e., the study population is affiliated with IMSS clinics and is therefore not representative of the general population. Second, we needed a sufficient sample size to ensure sufficient power in the hemoglobin and diet models. Third, the hemoglobin levels were obtained from clinical records, and it was not possible to access serum ferritin measurements to evaluate the etiology of anemia. However, 80% of cases of anemia in pregnancy are due to iron deficiency (45). The pre-gestational BMI was self-reported by the participants and may have produced an error in the estimates, which could lead to an underestimation of the association with maternal weight. When measuring diet through the 24-h recall (20), the information bias is significant since sometimes the participants do not remember or do not want to report food consumption, obtaining an under-reporting of the exposure variable. Covariates such as physical activity and sleep quality that affect energy balance were not measured and considered in the models. Diet was not assessed more frequently within the same trimester.

## 5 Conclusion

In this cohort, pregnant women have an excessive intake of UPF, particularly those with the double burden of malnutrition, pre-pregnancy obesity, and low levels of Hb. On average, UPF contributes to 27, 29, 26.5, and 31% of total calories, total sugar, saturated fat, and sodium, respectively. Urgent actions in the counseling since antenatal care regarding diet quality as high UPF consumption is associated with an increased risk of mortality from all causes, cardiovascular diseases, metabolic syndrome, overweight, obesity, irritable bowel syndrome, different types of cancer in adults (46), and increased weight gain in pregnancy (16), effects that can affect mother and their offspring.

## Data availability statement

The original contributions presented in the study are included in the article/supplementary material, further inquiries can be directed to the corresponding authors.

## Ethics statement

The studies involving humans were approved by The Research Ethics Committee of the National Institute of Public Health (#1281). The studies were conducted in accordance with the local legislation and institutional requirements. The participants provided their written informed consent to participate in this study.

## Author contributions

AG-A: Conceptualization, Formal analysis, Methodology, Visualization, Writing – original draft, Writing – review & editing. AC-M: Conceptualization, Formal analysis, Methodology, Validation, Visualization, Writing – original draft, Writing – review & editing. AC: Conceptualization, Formal analysis, Funding acquisition, Project administration, Resources, Supervision, Validation, Writing – original draft, Writing – review & editing. DC: Conceptualization, Funding acquisition, Resources, Supervision, Writing – original draft, Writing – review & editing. JM-P: Data curation, Validation, Writing – original draft, Writing – review & editing. LÁ-J: Resources, Writing – original draft, Writing – review & editing. IR-S: Data curation, Project administration, Validation, Writing – original draft, Writing – review & editing. JR: Investigation, Writing – original draft, Writing – review & editing. LG: Writing – original draft, Writing – review & editing. IB: Writing – original draft, Writing – review & editing. HL-F: Formal analysis, Methodology, Supervision, Validation, Writing – original draft, Writing – review & editing.
